# Superb Enhancement of Hydrogen Evolution in an Acidic Medium over a Cobalt Oxide Surface with Trace Incorporation of Rhodium Particles

**DOI:** 10.1021/acsomega.5c08740

**Published:** 2025-12-18

**Authors:** Kazi Hamidur Rashid, Mohammad Imran Hossain, Md Abdul Malek, Mohammad Afsar Uddin, Kentaro Aoki, Yuki Nagao, Nayan Ranjan Singha, Mostafizur Rahaman, Merajuddin Khan, Mohammad A. Hasnat

**Affiliations:** † Electrochemistry & Catalysis Research Laboratory (ECRL), Department of Chemistry, School of Physical Sciences, 113074 Shahjalal University of Science and Technology, Sylhet 3114, Bangladesh; ‡ Department of Chemistry and Biochemistry, 8063 The University of Alabama, Tuscaloosa, Alabama 35487, United States; § School of Materials Science, 12837 Japan Advanced Institute of Science and Technology, Nomi, Ishikawa 923-1292, Japan; ∥ Advanced Polymer Laboratory, Department of Polymer Science and Technology, 208295 Government College of Engineering and Leather Technology (Post-Graduate), Kolkata, West Bengal 700106, India; ⊥ Department of Chemistry, College of Science, 37850 King Saud University, P.O. Box 2455, Riyadh 11451, Saudi Arabia; # Instituto de Ciencia de Materiales de Madrid (ICMM), CSIC, C/Sor Juana Ines de la Cruz 3, Madrid 28049, Spain

## Abstract

Integration engineering is commonly used for the morphological development of electrocatalysts for promoting hydrogen evolution reactions (HER) due to their distinctive structures, whereas modifying the crystallinity can give the electrocatalyst unique properties that enhance its performance for HER. Herein, we successfully anchored rhodium (Rh) nanoparticles in Co_3_O_4_ thin film on a graphite (GP) surface. The obtained catalyst exhibits great HER catalytic performance in a 0.5 M H_2_SO_4_ medium. Interestingly, our findings show that the electrode prepared with 1-cycle (1c) electrodeposition of Rh onto Co_3_O_4_@GP (Rh1c/Co_3_O_4_@GP) surpasses the HER performance of multiple cycles of Rh deposition onto Co_3_O_4_@GP or even a 10-cycle Rh electrodeposition Rh10c@GP electrode in the absence of Co_3_O_4_, indicating the crucial role of well-engineered Rh deposition and Co_3_O_4_ in the overall HER activity. For acidic HER, the catalyst required overpotentials of only 57 mV to deliver a current density of 10 mA cm^–2^, which is nearly identical to the value by commercial Pt/C. Notably, the Rh1c/Co_3_O_4_@GP shows almost no degradation even after 8 h of stability experiments. The synergistic integration of Co_3_O_4_@GP with Rh enhances the availability of active sites and improves the intrinsic catalytic activity, as a result showing outstanding HER activity and higher stability. The structural and surface characteristics of the Rh-doped Co_3_O_4_-modified graphite electrodes were examined by using X-ray diffraction (XRD), energy-dispersive X-ray spectroscopy (EDX), field emission scanning electron microscopy (FESEM), and X-ray photoelectron spectroscopy (XPS).

## Graphical abstract

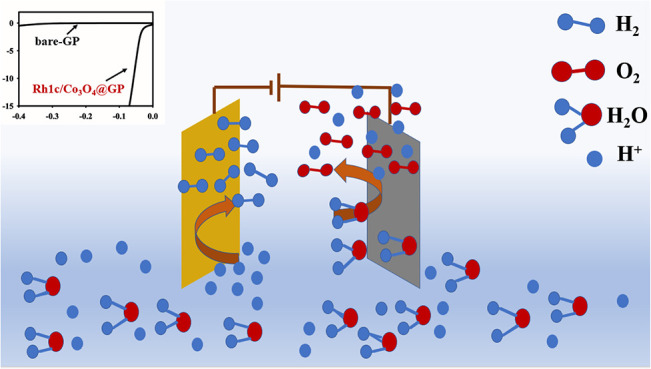

## 1 Introduction

With the steady rise in the global population, the need for resources and energy is increasing at an exponential pace. Fossil fuels serve as the primary energy source; however, their usage leads to the warming of the Earth, the release of greenhouse pollutants, acidified rainfall, and various other environmental problems.^1^ Therefore, it is essential to explore and consider alternative energy sources. Renewable energy sources such as solar, wind, biomass, and hydrogen are options that can help to reduce greenhouse gas release and fight global warming.^2^ Hydrogen energy has become a sustainable and ecofriendly option to fossil fuels in the past few years. Hydrogen, one of the most abundant elements on Earth, has the capability to substitute fossil fuels in the future, regardless of the method used for its production.^3^ It serves as a sustainable power source that helps reduce environmental impact while offering cleaner energy production.^4^ Hydrogen consists solely of a proton and an electron. It is also scent-free and transparent and has a lower density than air. Additionally, its mass-based energy density is approximately seven times greater than that of fossil fuels.^5^

There are various approaches of producing hydrogen, including methane reforming from fossil fuels, utilizing electrical decomposition by water splitting, microbial breakdown of organic matter or water, extreme heat-assisted separation of water, sunlight-driven cleavage of water, and light-induced electrochemical dissociation.^6^ Among the different hydrogen generation techniques, water splitting carried out through the electrochemical method emerges as one of the most promising and environmentally sustainable options. Unlike thermochemical processes such as steam methane reforming, which release large amounts of carbon dioxide, electrolysis can generate hydrogen without producing greenhouse gases, especially when it is powered by renewable energy sources. In comparison of electrolysis with biological and photochemical methods, we can say that electrolysis offers greater scalability, higher efficiency, and more advanced technological development, which makes it suitable for real-world problems and applications. Furthermore, its compatibility with solar and wind power places it as a key strategy for large-scale green hydrogen production, contributing to global environmental objectives toward carbon neutrality and sustainable energy transition.^7^ During the electrochemical hydrogen evolution reaction (HER), water splitting involves the flow of electrons, the migration of protons, and the formation of gas bubbles, leading to the formation of hydrogen gas (as shown in eq 1).

1 $$H_{2}O_{\left(l\right)}+{H_{3}O^{+}}_{\left(\mathrm{aq}\right)}\to H_{2(g)}+O_{2(g)}+H^{+}$$

Electrochemical water splitting for (HER) offers a suitable method for producing hydrogen due to its higher effectiveness, minimal power usage, and adaptability with sustainable energy solutions.^8–14^ The HER occurs through one of two pathways: Volmer–Heyrovsky or Volmer–Tafel. The overall reaction behavior can be interpreted from the Tafel slope, where a slope of ∼120 mV dec^–1^ indicates that the Volmer step limits the reaction rate, ∼40 mV dec^–1^ suggests that the Heyrovsky step serves as RDS, and ∼30 mV dec^–1^ highlights the Tafel recombination step, which is mentioned in eq 6. Therefore, a lower Tafel slope suggests accelerated proton adsorption and recombination kinetics, often resulting from improved electron transfer and optimal hydrogen binding energy on the catalyst surface.^15^

Noble metals such as platinum, together with their metallic alloys, are acknowledged as highly effective electrocatalysts for the HER, as they facilitate the transformation of protons and electrons into hydrogen at a minimal overpotential.^16–19^ However, the limited availability and high expense of commercially viable noble metals hinder their practical use in the HER.^20^ Therefore, developing alternative catalysts with comparable efficiency, abundant availability, affordability, and durability is a crucial advancement toward a sustainable hydrogen economy.^21,22^ As a result, considerable attention has been directed toward developing nonprecious metal catalysts such as metal carbides, phosphides, and sulfides, along with doped and heterostructured materials.^3,20–21,22,23–32,33,34,35^ Recent investigations reveal that incorporating small quantities of noble metals into transition metal systems or designing thin-film architectures can markedly enhance HER performance.^34,35^ For example, Zou et al. synthesized a noble metal-based thin film RhRuPtPdIr by using atomic layer deposition (ALD) followed by electrical Joule heating, demonstrating outstanding HER activity with a minimal overpotential of 13 mV at 10 mA cm^–2^ in an acidic environment. The improved performance was mainly due to the improvement of a noncrystalline surface coating and changes in the electronic properties, both of which facilitated better hydrogen adsorption and desorption.^36^ Similarly, cobalt-doped TiO_2_ nanorods showed enhanced HER activity in alkaline conditions. This resulted from the incorporation of Co and the generation of oxygen defect sites. The optimized system experienced an overpotential of 78 mV at 10 mA cm^–2^, which exhibits high stability. where theoretical studies revealed that Co incorporation minimized the energy barriers for water dissociation and hydrogen intermediate release.^37^ In a different study, a researcher developed Ni/Fe_3_O_4_ nanoparticles with tailored heterostructures that optimized the hydrogen adsorption behavior. This catalyst showcased an impressively low overpotential of 46 mV at a current density of 10 mA cm^–2^ and a Tafel slope of 58 mV dec^–1^, highlighting the effectiveness of heterostructure engineering in improving HER performance.^38^ Moreover, a different researcher developed a silver nanowire that is incorporated with MoO_3_–Co­(OH)_2_, demonstrating superior HER performance relative to their individual components. The synergistic interface facilitated Gibbs free energy nearly at zero for the adsorption of hydrogen (Δ*G*_H_*), which indicates favorable catalytic kinetics.^39^ In another investigation, density functional theory (DFT) analysis was used to examine Mn-doped MoS_2_ monolayer, which showed that Mn incorporation activated the basal planes and facilitated the Volmer–Heyrovsky pathway by lowering the energy barriers from 10.34 to 10.79 kcal/mol, thereby improving HER kinetics and decreasing the Tafel slope.^40^ Additionally, a different scientist investigated Pd/SnTe heterostructures, where the presence of topological surface states (TSSs) in SnTe significantly boosted HER performance beyond that of pure Pd thin films and commercial Pt foils by facilitating electron transfer and modulating Pd–H binding strength.^41^

Although notable advancement has been achieved, research on improving the HER performance of transition metal oxides (TMOs) through minor incorporation of noble metals remains scarce. TMOs like Co_3_O_4_ are commonly employed in various electrocatalytic reactions such as NRR, ORR, OER, CO_2_RR, and HER due to their rich adaptability and natural abundance.^37^ Rhodium (Rh) exhibits higher catalytic activity, ideal Gibbs free energy (Δ*G*_H_**), and strong electrochemical stability for HER in both acidic and alkaline media.^42–44^ Its incorporation into transition metal oxides enhances active site dispersion, charge transfer, and hydrogen adsorption energetics, leading to superior HER performance. In the ongoing study, we describe the fabrication of a Rh-modified cobalt oxide (CoO_*x*_) electrocatalyst (Rh1c/Co_3_O_4_@GP), where trace Rh incorporation optimizes the surface electronic environment and improves hydrogen adsorption kinetics.^38^ Cobalt was chosen due to its earth abundance, low toxicity, and unique redox properties, while graphite provides a stable conductive support.^40,41^ Rhodium was added to our composite to enhance the interaction of reaction intermediates with the catalyst due to its partially filled 4d orbitals. This addition strengthens resistance when the reaction conditions are extreme, which provides greater electrochemical stability. Moreover, the CoO_*x*_-Rh composite shows the highest H_2_ selectivity due to Co reduction, which is enhanced by Rh insertion.^38,41,45^

Through the use of ultralow Rh loading (Rh1c/Co_3_O_4_@GP), our system maximizes atomic utilization and enhances the electronic structure of Co_3_O_4_, while the synergistic interaction between Rh single atoms and Co in the composite improves electron transport and catalytic stability. This combination enables superior HER performance with very low overpotential, excellent stability, and minimized noble metal usage, which offers a cost-effective strategy for efficient hydrogen evolution, which makes it to comparable to Pt/C in an acidic medium.

## 2 Experimental Section

### 2.1 Chemicals and Reagents

For this research, chemical reagents were of analytical-grade and purchased with maximum purity. Rhodium­(III) chloride (RhCl_3_·*n*H_2_O), cobalt­(II) sulfate (CoSO_4_), and sulfuric acid (H_2_SO_4_) were supplied by Sigma-Aldrich, based in St. Louis, MO, USA. To guarantee the elimination of any contaminants that might interfere with the experimental outcomes, Milli-Q ultrapure water was used in the preparation of all solutions.

### 2.2 Electrodes, Electrochemical Cells, and Instrumentation

The electrochemical investigations were conducted in a single-compartment three-electrode cell using a CHI-660 potentiostat (CHI Instruments, USA) and an Autolab Potentiostat (PGSTAT128N, Netherlands) in a standard three-electrode configuration. The support electrode material was graphite, which underwent eight cycles of cobalt layering process, followed by a thin coating of rhodium in trace amounts achieving a final geometric area of around 0.283 cm^2^, whereas a Pt wire was used as the counter electrode and an Ag/AgCl (3 M KCl) electrode was employed as the reference electrode.

The structural and morphological properties of the synthesized materials were analyzed. PXRD measurements were carried out on a Rigaku SmartLab diffractometer with Cu Kα irradiation (λ = 1.5406 Å). The morphology was examined with FESEM (JSM-7610F, Japan), while the elemental composition was analyzed via EDX using a TM3030Plus miniscope (Hitachi Ltd.). XPS spectra were obtained by using a delay-line detector (DLD) spectrometer (Kratos Axis Ultra; Kratos Analytical Ltd.) with an Al Kα radiation source (1486.6 eV).

### 2.3 Electrode Fabrication

A regular drawing pencil (PERRA PHOENIX-12B) was used to prepare the graphite electrode (GP). The pencil was shaped into an electrode similar to a conventional laboratory electrode, leaving the graphite core exposed, while the rest was sealed with a water-resistant, biodegradable plastic coating. Before use, the exposed graphite surface was thoroughly cleaned with Milli-Q ultrapure water (17.3 MΩ cm resistivity) and 1 M H_2_SO_4_, followed by polishing with 120-grit sandpaper and a carbon pad. The electrodes were then rinsed again with ultrapure water and dried at room temperature. For electrodeposition, cobalt was first deposited onto the GP surface from an aqueous 0.1 M CoSO_4_·7H_2_O solution without any supporting electrolyte. The deposition was performed by cyclic voltammetry within the potential range of −1.1 to +0.5 V vs Ag/AgCl for eight cycles at a scan rate of 0.1 V s^–1^. Several Co-modified GP electrodes were prepared in this way. Subsequently, rhodium was electrodeposited on the Co/GP electrodes from a 0.05 M RhCl_3_·*n*H_2_O solution by repeatedly cycling the potential between 0 and −0.25 V vs Ag/AgCl at the same scan rate, varying the number of deposition cycles to control Rh loading. The resulting electrodes were designated as Rh1c/Co_3_O_4_@GP, Rh5c/Co_3_O_4_@GP, Rh7c/Co_3_O_4_@GP and Rh10c/Co_3_O_4_@GP, corresponding to 1, 5,7, and 10 deposition cycles, respectively. All electrochemical experiments, including HER activity, were conducted in 0.5 M H_2_SO_4_ solution.

All potential values were initially recorded against the Ag/AgCl reference electrode and later transformed into the reversible hydrogen electrode (RHE) scale by using eq 2.

2 $$E_{\mathrm{RHE}}=E_{\mathrm{Ag}/\mathrm{Ag}\mathrm{Cl}}+0.197+0.059\times \mathrm{pH}$$

The overpotential (η_10_) needed to reach a current density of 10 mA cm^–2^ was used as a standard metric to compare the HER performance, as this current density is commonly adopted to represent the practical benchmark for hydrogen generation efficiency.

All solutions were made by using ultrapure water. Electrochemical measurements were performed using a CHI-660 potentiostat (CHI Instruments, USA) and an Autolab PGSTAT128N (Metrohm, Netherlands). The geometric area of the graphite electrode was used to calculate the current densities.

## 3 Results and Discussion

### 3.1 Open Circuit Potential (OCP)

The open circuit potential (OCP) serves as a foundational indicator for assessing electrode behavior, particularly as it describes the inherent potential on an electrode surface when Faradaic progression occurs.^46^ In our study, we measured the OCP values for the developed electrodes: bare-GP, Co_3_O_4_@GP, Rh10c@GP, Rh1c@GP, Rh10c/Co_3_O_4_@GP and Rh1c/Co_3_O_4_@GP. The bare-GP electrode exhibited an OCP of 0.72 V vs RHE. When 10c of Rh was employed (Rh10c@GP), the OCP dropped to 0.60 V indicating a more reducing surface than the bare GP as shown in Figure 1. Notably, the Rh1c/Co_3_O_4_@GP electrode exhibited a lower open-circuit potential of 0.36 V. This negative shift in the OCP suggests that Rh incorporation promotes more effective movement of charge at the electrode–electrolyte boundary. The Rh and Co_3_O_4_ interact strongly at their interface, which includes metal-oxide bonding, and simultaneously improves the surface electronic structure and reduces interfacial resistance. As a result, the HER performance becomes much better.^47,48^

**Figure 1:**
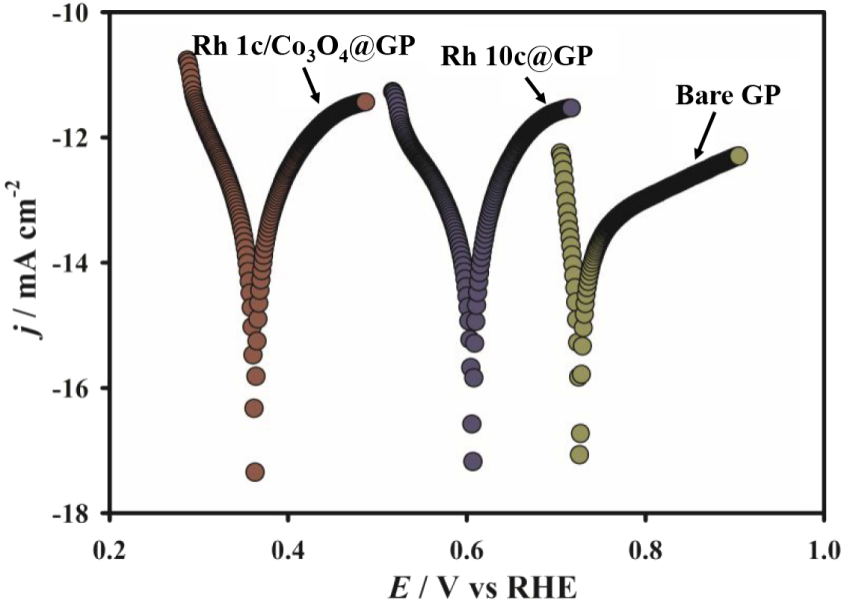
Linear polarization measurements were carried out in 0.5 M H_2_SO_4_ solution using bare GP, Rh10c@GP, and Rh1c/Co_3_O_4_@GP electrodes at a scan rate of 0.1 V s^–1^.

The shifting in the OCP values implies that the modified surface is becoming more electron-rich, which is advantageous for HER activity. Since the initial step of HER entails the binding of hydrogen ions (H^+^) onto the surface of the electrode, a more negative OCP suggests a more favorable environment for this process. Specifically, the significantly lower OCP observed for Rh1c/Co_3_O_4_@GP compared to both GP and Rh10cGP suggests that this composite structure creates a more reducing surface, likely promoting faster proton adsorption and enhanced HER kinetics. Thus, the trend in the OCP supports the idea that Rh1c/Co_3_O_4_@GP provides a superior interfacial condition for HER compared to other electrode configurations examined in this work.

### 3.2 HER Catalysis

In our study, the HER performance was rigorously evaluated for several electrodes, including bare graphite (GP), Co_3_O_4_-modified GP (Co_3_O_4_@GP), Rh-deposited GP (Rh1c@GP and Rh10c@GP), and their composite forms (Rh1c/Co_3_O_4_@GP and Rh10c/Co_3_O_4_@GP), alongside commercial Pt/C as a reference catalyst—all examined under the same experimental conditions. As illustrated in Figure 2A, the bare GP electrode exhibited poor HER activity, requiring a high overpotential of 717 mV to achieve a current density of 10 mA cm^–2^. This clearly confirms the catalytic inertness of the GP substrate and suggests that any improved HER performance in the composite systems arises from the active materials deposited onto the GP surface. Due to modification of GP with a thin layer of Co_3_O_4_, a slight enhancement in HER activity was observed, which decreased the required overpotential to 595 mV at 10 mA cm^–2^. However, this modest improvement indicates that Co_3_O_4_ alone does not significantly boost the catalytic efficiency. Further enhancement was achieved by depositing Rh nanoparticles on GP. Both Rh1c@GP and Rh10c@GP showed substantial improvements in HER activity, necessitating overpotentials of just 228 mV and 48 mV, respectively, to reach 10 mA cm^–2^. These results highlight Rh’s strong intrinsic activity toward HER. To explore potential synergistic effects, we combined Rh and Co_3_O_4_ on the GP surface. The electrodes prepared with different Rh deposition cycles on the Co_3_O_4_/GP surface were next tested for HER activity as shown in Figure 2B. It is seen that the highest negative overpotential (256 mV) @ 10 mA cm^–2^ current is observed while Rh10c/Co_3_O_4_@GP was used, which was prepared with 10 Rh deposition cycles. The overpotential gradually shifted to positive values as the number of Rh deposition cycles was reduced. The least overpotential 44 mV @10 mA cm^–2^ current is seen with the electrode Rh1c/Co_3_O_4_@GP, which was prepared with only a single Rh deposition cycle. Note that the catalytic efficiency of Co_3_O_4_ toward HER arises from its unique spinel structure and the combined presence of Co^2+^/Co^3+^ oxidation states, which promote rapid electron transport and reversible redox activity. Moreover, modification of such as nanostructuring, elemental doping, and forming composites further enhance its activity by exposing more active sites and improving electrical conductivity.^49^ Meanwhile, rhodium exhibits strong HER activity because its hydrogen binding energy is close to the optimal range, enabling efficient adsorption and desorption of hydrogen. This behavior is quite similar to the Sabatier principle and ensures better catalytic performance.^50^ Interestingly, the Rh10c/Co_3_O_4_@GP composite delivered a less favorable performance than expected, with a higher overpotential of 256 mV at 10 mA cm^–2^. The reason behind this low activity is likely due to excessive Rh loading, which may have caused nanoparticle aggregation or blocked active sites. As a result, this reduced the catalytic performance.

**Figure 2:**
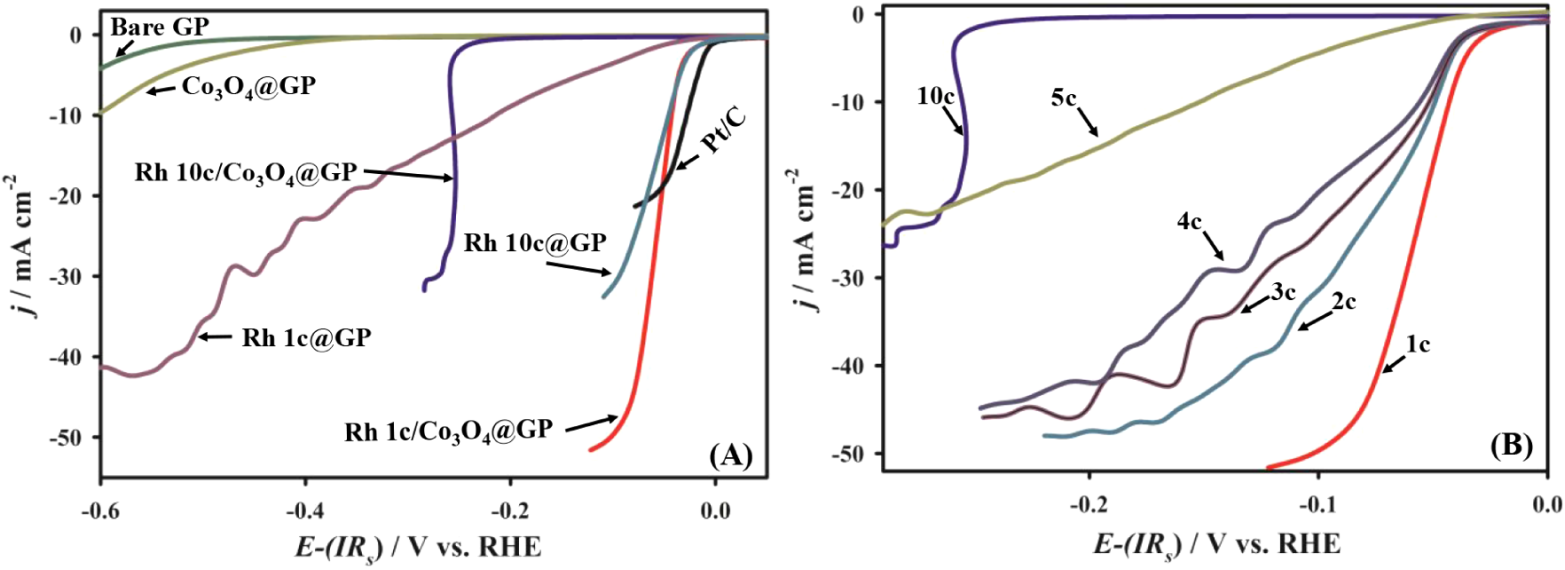
(A) Linear sweep voltammetry (LSV) curves of bare GP, Co_3_O_4_@GP, Rh1c@GP, Rh10c@GP, Rh10c/Co_3_O_4_@GP, and Rh1c/Co_3_O_4_@GP electrodes. (B) Cycle optimization of Rh on Co_3_O_4_@GP surface for HER activity recorded in 0.5 M H_2_SO_4_. Measurements were conducted at a scan rate of 0.01 V s^–1^.

In contrast, the Rh1c/Co_3_O_4_@GP composite showed exceptional HER activity with an overpotential of just 44 mV at a current density of 10 mA cm^–2^, which is almost equivalent to that of standard Pt/C. This result underscores the critical role of both optimal Rh loading and the synergistic interface between Rh and Co_3_O_4_ in enhancing HER kinetics. These findings suggest that at low Rh loading (Rh1c@GP), the Rh atoms are finely dispersed as tiny clusters or single sites, leading to lower conductivity and weaker HER activity than Rh10c@GP. Upon Co_3_O_4_ addition, Rh–O–Co interfaces that improve charge transfer through the Co^2+^/Co^3+^ redox pair, and trace Rh incorporation effectively tunes the electronic structure of Co_3_O_4_ and maximizes active interfacial sites, whereas excess Rh reduces the metal-oxide coupling and blocks active sites, leading to reduced HER activity.^51–53^

### 3.3 HER Kinetics

Next, Tafel measurements were carried out to understand the relative electrokinetic activities of Rh10c/Co_3_O_4_@GP and Rh1c/Co_3_O_4_@GP electrodes, to compare the effect of high and low Rh loading over Co_3_O_4_@GP substrate, using eq 3.^54^

3 $$\log\left(j\right)=\log\left(j_{k}\right)+b_{T}\left(E-E^{o}\right)$$

Here, *E* ^o^ indicates the standard potential of the electrode, *E* refers to the potential applied to the system (vs RHE), *j_k_* = *nFCK* ^o^ denotes the exchange current density at *E* = *E* ^o^. The term *j* corresponds to the measured current density, while *b*_T_ refers to the Tafel slope. It is well-established that most of the reactions proceed via multi-electron transfer mechanisms. Specifically, for the HER process in an acidic medium, the water reduction involves the subsequent steps (eqs 4–6), where any of these steps could be the rate-determining step (RDS).

4 $${H_{3}O^{+}}_{\left(\mathrm{aq}\right)}+1e^{-}\to H_{\mathrm{ad}}\;\left(\mathrm{Volmer}\;\mathrm{step}\right)$$

5 $$H_{\mathrm{ad}}+{H_{3}O^{+}}_{\left(\mathrm{aq}\right)}+1e^{-}\to H_{2(g)}+H_{2}O_{\left(l\right)}\;\left(\mathrm{Heyrovsky}\;\mathrm{step}\right)$$

6 $$H_{\mathrm{ad}}+H_{\mathrm{ad}}\to H_{2(g)}\;\left(\mathrm{Tafet}\;\mathrm{step}\right)$$

In the initial stage, a proton is reduced to an adsorbed hydrogen atom, a process known as the Volmer step (eq 4). In the subsequent step, the adsorbed hydrogen atom undergoes further reduction by reacting with another proton to form molecular hydrogen, and this is known as the Heyrovsky step (eq 5). Alternatively, molecular hydrogen can also be generated through the combination of two hydrogen atoms attached to the surface, which is termed the Tafel step (eq 6).

Tafel slope analysis provides further insight into the HER kinetics of the investigated electrodes, as shown in Figure 4. It is crucial to recognize that if the RDS carries a charge transfer coefficient of 0.5, the Tafel slope (*b*_T_) can be written as $b_{T}=\frac{2.303RT}{\left(n+0.5\right)F}$ , where *n* represents the number of electrons moving during the reaction ahead of the RDS. Meanwhile, if no electron transfer occurs before the RDS (*n* = 0), the Tafel slope is expected to be approximately 120 mV/dec. If a single electron transfer takes place before the RDS (*n* = 1), the slope would be around 40 mV/dec. Conversely, if two electrons are transferred before the RDS and the subsequent step is purely chemical (like eq 6), the Tafel slope becomes $b_{T}=\frac{2.303RT}{2F}=30\mathrm{mV}{\mathrm{dec}}^{-1}$ .^55^

Figure 3 displays the Tafel plots and their respective slope values for different electrodes. Among the electrodes employed, the bare GP electrode exhibited the poorest catalytic activity, as reflected by a relatively high Tafel slope of 115 mV dec^–1^. Surprisingly, when the Co_3_O_4_ is incorporated onto the GP surface, it results in an even higher slope of 199 mV dec^–1^, indicating further sluggish HER kinetics. This observation suggests that Co_3_O_4_ alone not only fails to enhance but also decreases HER activity on the GP surface, owing to its low intrinsic electrical conductivity and the few active sites available for the reaction. A significant shift in performance is observed with the incorporation of Rh. Meanwhile, the composites Rh1c@GP and Rh10c@GP demonstrated marked improvements, with Tafel slopes of 88 mV dec^–1^ and 69 mV dec^–1^, respectively, reflecting accelerated reaction kinetics because of the existence of catalytically active Rh nanoparticles. Notably, the Rh1c/Co_3_O_4_@GP composite achieved the lowest Tafel slope of 39 mV dec^–1^, underscoring a highly efficient HER process and follows a Heyrovsky mechanism where a proton (H^+^) from the solution combines with a hydrogen atom (H_ad_) attached to the electrode surface and an electron (e^–^) to form molecular hydrogen (H_2_), which limits the reaction rate. This observed performance is similar to that of commercial Pt/C (36 mV dec^–1^). This improvement arises from the synergistic interaction between Rh and Co_3_O_4_, where Rh provides highly active sites with near-optimal hydrogen binding energy, while Co_3_O_4_ promotes efficient charge transfer and stabilizes the catalytic interface. The combination facilitates faster Volmer–Heyrovsky processes, minimizing the activation barrier for H–H recombination in eq 6), thereby resulting in a reduced Tafel slope. On the other hand, due to higher deposition of Rh content, the Rh10c/Co_3_O_4_@GP electrode exhibited a substantially higher Tafel slope (93 mV dec^–1^) compared to the Rh1c/Co_3_O_4_@GP (39 mV dec^–1^), indicating a declined HER activity. This deterioration can be attributed to excess Rh deposition, which may cause agglomeration or coverage of active sites, ultimately limiting the availability of catalytic surfaces and hindering charge transfer.

**Figure 3:**
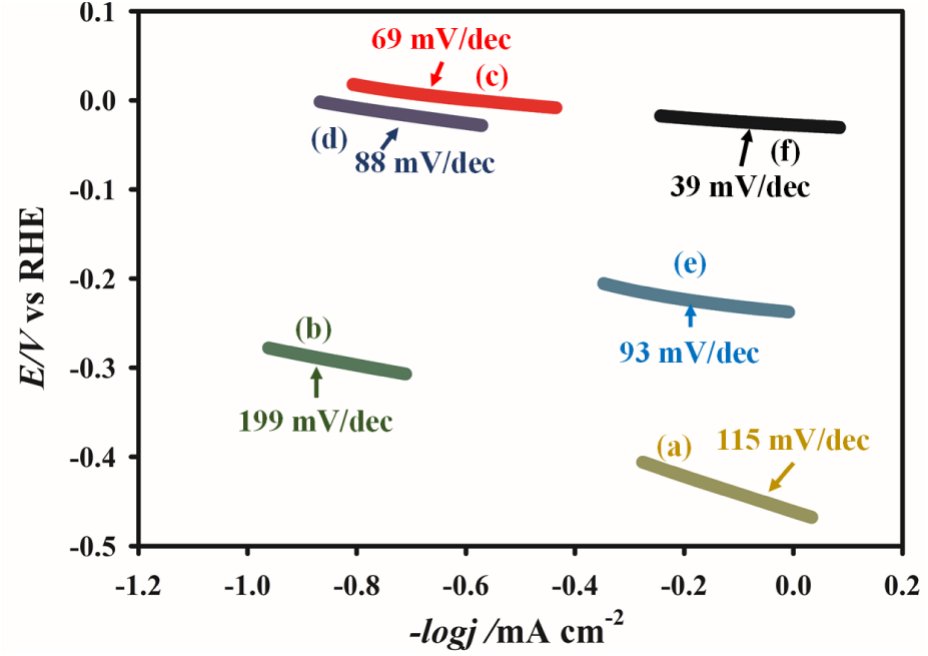
Tafel plots of (a) bare GP, (b) Co_3_O_4_@GP, (c) Rh10c@GP, (d) Rh1c@GP, (e) Rh10c/Co_3_O_4_@GP, and (f) Rh1c/Co_3_O_4_@GP recorded in 0.5 M H_2_SO_4_ electrolyte for the HER at a scan rate of 0.01 V s^–1^.

### 3.4 ECSA Estimation

The electrochemically active surface area (ECSA) is essential for evaluating the intrinsic catalytic performance of a modified electrode. It allows for a more precise comparison between different catalysts by offering insight into the actual active interface available for electrochemical reactions and is also essential for gauging the true activity of catalytic materials.^56^ A widely adopted approach for estimating ECSA involves evaluating the double-layer charge storage capacity (*C*_dl_), which represents the nonfaradic current response in a potential region free from redox activity. The connection between the capacitive current (*i*) and scan rate (d*V*/d*t*) is expressed in eq 7.

7 $$i=C_{\mathrm{dl}}\frac{dV}{dt}$$

ECSAs were estimated for bare-GP, Co_3_O_4_@GP, Rh10c@GP, and Rh1c/Co_3_O_4_@GP in 0.5 N H_2_SO_4_ solution using the technique. The capacitive current response was plotted against the scan rate, using collected data from CV measurements at various scan rates, as illustrated in Figure S3. The slope of these plots provided the *C*_dl_ values: 1.6, 0.9, 0.8, and 1.1 mF cm^–2^ for bare-GP, Co_3_O_4_@GP, Rh10c@GP, and Rh1c/Co_3_O_4_@GP, respectively. Afterward, using the known geometric surface area of the bare-GP electrode (0.125 cm^2^), the ECSA of the modified electrodes was determined through eq 8.

8 $${\mathrm{ECSA}}_{\mathrm{modified}}=\frac{C_{\mathrm{dl}}\left(\mathrm{modified}\right)}{C_{\mathrm{dl}}\left(\mathrm{bare}\;\mathrm{GP}\right)}\times A_{\mathrm{bare}\;\mathrm{GP}}$$

From the ECSA data tabulated in Table 1, the deposition of Co_3_O_4_ increased the ECSA to 0.222 cm^2^, likely due to the porous and high-surface nature of the cobalt oxide layer. However, when a single Rh deposition cycle was applied over Co_3_O_4_@GP, the ECSA moderately decreased to 0.1945 cm^2^, where a balance between coverage and exposure of active sites was achieved between Co–Rh composite. Furthermore, its suitability for HER is highlighted as it steadily maintains a higher reactive area compared to Rh10c@GP.

From the calculated ECSA and the slopes of current vs scan rate, the number of electrochemically active sites (*m*) was calculated using the following expression shown as eq 9.

9 $$i=\frac{n^{2}F^{2}{\mathrm{ESCA}}_{\left(\mathrm{modified}\right)}m}{4RT}\times v$$

The values of “*m*” obtained were: 13.63 × 10^–9^, 4.32 × 10^–9^, 6.02 × 10^–9^, and 6.02 × 10^–9^ mol cm^–2^ for bare-GP, Co_3_O_4_@GP, Rh10c@GP, and Rh1c/Co_3_O_4_@GP, respectively.

**Figure 4:**
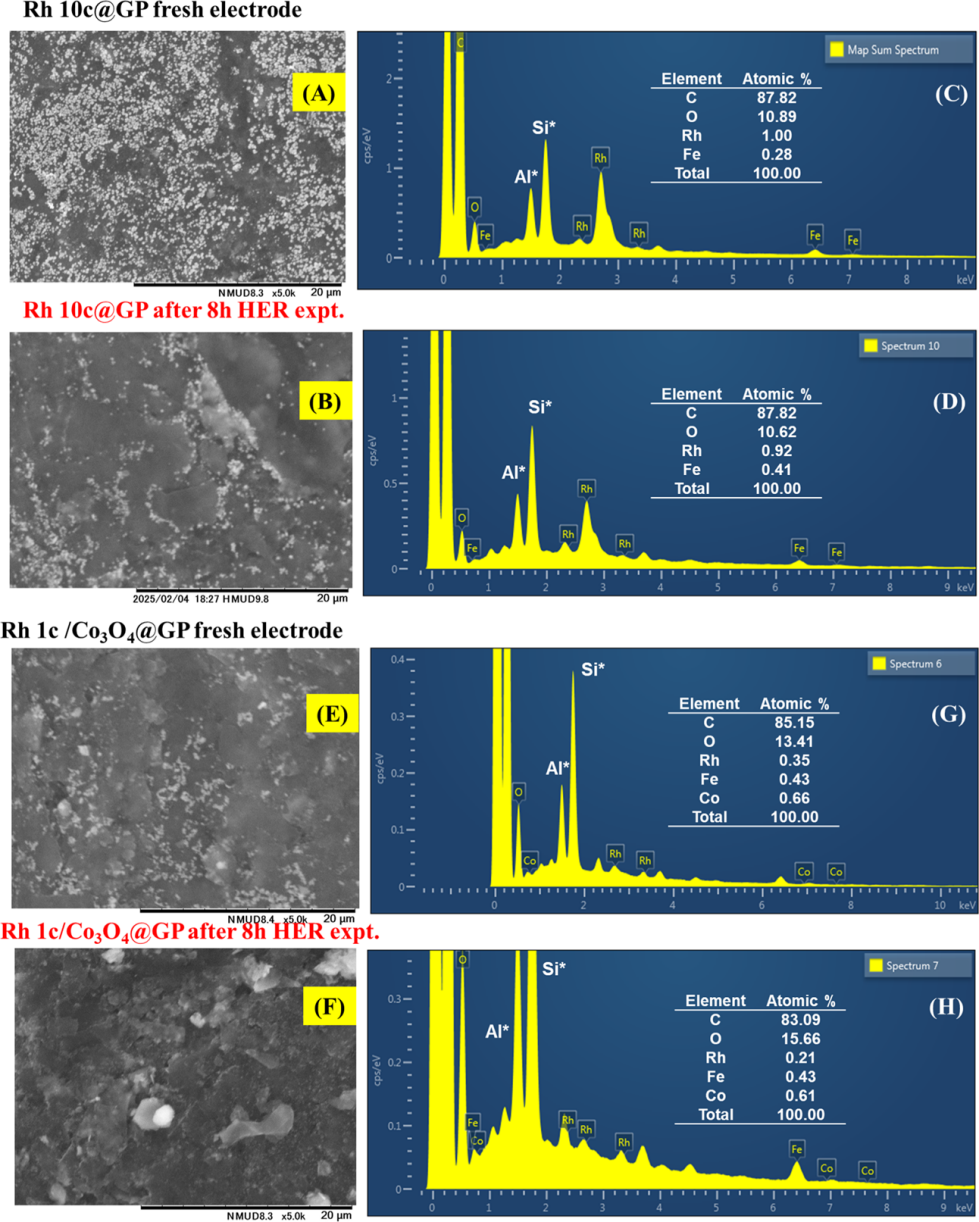
SEM images and EDS spectra of (A–D) Rh10c@GP and (E–H) Rh1c/Co_3_O_4_@GP electrodes before and after HER experiments, respectively.

**Table 1:** Evaluation of the ESCA of Various Catalysts Depending on Their *C*_dl_ Value and the Related TOF Was Determined from These ESCAs

Catalysts	Bare-GP	Co_3_O_4_@GP	Rh1c Co_3_O_4_@GP
ECSA (cm^2^)	—	0.2220	0.1415
TOF (s^–1^)	2.5 × 10^–4^	1.35 × 10^–3^	8.4 × 10^–4^

### 3.5 TOF Analysis

The turnover frequency (TOF) describes the crucial property of an electrocatalyst’s performance for a specific reaction. It is defined as the number of moles of product formed per mole of active site per unit time. Therefore, the TOF highlights the catalytic activity of the material. The catalytic activity increases as the TOF value increases.^56^ The TOF was calculated using the following eq 10.

10 $$\mathrm{TOF}=\frac{jA}{2Fm}$$

TOF data obtained at *j* = 5 mA cm^–2^ and tabulated in Table 1 revealed that GP and Co_3_O_4_@GP exhibited a TOF of 2.5 × 10^–4^ s^–1^ and 1.35 × 10^–3^ s^–1^, respectively representing their limited intrinsic activity. Meanwhile, incorporation of a tiny amount of Rh on Co_3_O_4_@GP surface resulted in the electrode Rh1c/Co_3_O_4_@GP to achieve the highest TOF of 8.4 × 10^–4^ s^–1^ validating the synergy between minimal Rh loading and the Co_3_O_4_ scaffold.

### 3.6 Surface Analyses

Stability of a material is an important factor to establish it as a catalyst to execute a specific chemical process. Thus, to understand the morphological and compositional differences of the two most active fabricated electrodes, those were prepared in the absence and presence of Co_3_O_4_ particles (i.e., Rh10c@GP and Rh1c/Co_3_O_4_@GP, respectively), SEM and EDS analyses were performed first for both electrodes before and after 8 h of HER experiments.

The results are presented in Figure 4. The SEM images of Rh10c@GP (Figure 4A,B) reveal a surface densely populated with fine particles, indicating possible agglomeration and an uneven distribution of Rh particles. Although the surface appears rough, it lacks the well-defined structural features typically associated with efficient HER activity. This irregular morphology likely leads to a decrease in the available active sites and hampers effective charge transfer. These observations align with the EDS data shown in Figure 4C,D, which confirm the presence of 87.82 at. % C, 10.58 at. % O, and 1.00 at. % Rh, along with trace amounts of Fe originating from commercial pencil graphite. Interestingly, the Rh content remains nearly unchanged even after the prolonged HER experiment, suggesting good elemental stability. However, the overall morphology and poor surface structure likely undermine its catalytic performance due to blocked active sites and limited synergy with other elements.

In contrast, the Rh1c/Co_3_O_4_@GP electrode (Figure 4E,F) exhibits a more uniform and well-organized surface morphology before and after HER operation. The SEM images show evenly distributed particles and larger, brighter clusters, which are attributed to the successfully deposited Co_3_O_4_ nanoparticles. In comparison to the Rh10c@GP, the surface of Rh1c/Co_3_O_4_@GP appears more porous and electrochemically accessible—characteristics that are highly favorable for HER. The EDS spectra in Figure 4G,H support these morphological findings, showing elemental compositions ranging from 83.10 to 85.15 at. % C, 13.41–15.56 at. % O, 0.21–0.43 at. % Rh, and 0.65–1.01 at. % Co. Despite the significantly lower Rh content, this electrode outperforms the Rh10c@GP sample. The presence of Co_3_O_4_ not only confirms successful codeposition but also suggests a strong synergistic interaction with Rh, enhancing the overall catalytic activity. The higher oxygen content also reinforces the presence of metal oxides, contributing to better HER performance through improved H^+^ adsorption and electron transfer kinetics.

Powder X-ray diffraction (PXRD) was used to examine the crystal structure and phase composition of the Rh10c-@GP and Rh1c/Co_3_O_4_-@GP electrodes before and after HER stability tests. Diffraction peaks shown in Figure 5A originated from metallic Rh on the electrode surface found at around 2θ ≈ 40.0°, 47.5°, and 69.2°, closely match the values reported in previous studies.^57^ According to previously reported literature, a broad diffraction peak should have been observed at 2θ ≈ 26.5°, related to the (002) plane typical of graphitic carbon.^58^ However, in the case of the Rh10c @ GP modified sample, this peak was not visible, likely due to the formation of a relatively thick Rh layer that masked the underlying graphitic signal. A comparison of the diffraction patterns before and after the 8 h HER test confirms the structural robustness of the electrode, with no notable phase transitions detected.

**Figure 5:**
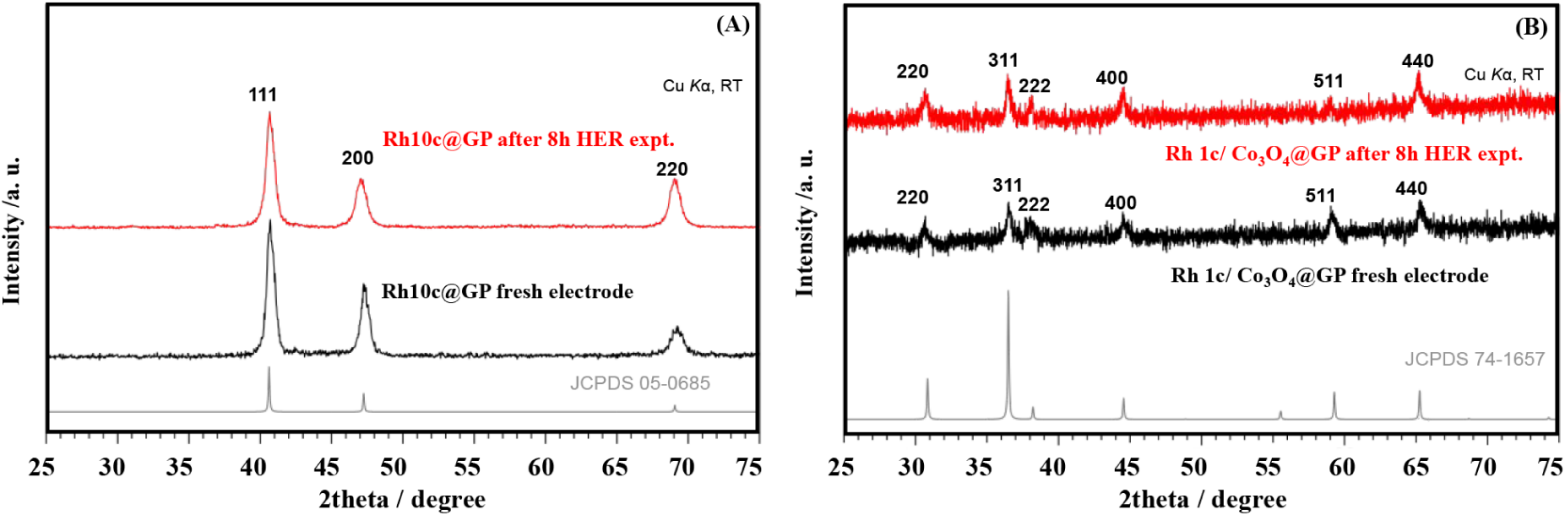
XRD patterns of (A) Rh10c-@GP and (B) Rh1c/Co_3_O_4_-@GP electrode surfaces before and after the stability test.

In contrast, the XRD pattern for Rh1c/Co_3_O_4_@GP as illustrated in Figure 5B, the fresh electrode with black color displays sharp peaks at 2θ ≈ 31.2°, 36.8°, 38.5°, 44.8°, 59.3°, and 65.2°, attributed to the (220), (311), (222), (400), (511), and (440) planes of Co_3_O_4_, which closely align with the standard reference (JCPDS No. 74-1657), demonstrating the successful formation of Co_3_O_4_ in its crystalline form as the major phase.^59^ No clear diffraction peaks corresponding to Rh and GP were detected, which may be due to their minimal content, amorphous character, or peak overlapping with Co_3_O_4_ peaks. After an 8 h HER expt., the primary diffraction peaks remain at their previous position and intensity, indicating the structural stability of Co_3_O_4_ under HER operation.^57^

The X-ray photoelectron spectroscopy (XPS) was used to investigate the surface chemistry of Rh10c@GP and Rh1c/Co_3_O_4_@GP composites before and after 8 h HER experiments. In the case of the Rh10c@GP electrode shown in Figure 6A, the Rh 3d peaks were observed at ∼307.18 eV and ∼311.94 eV in the fresh sample and at ∼307.84 eV and ∼311.61 eV after the HER experiment, corresponding to Rh 3d_5/2_ and 3d_3/2_, respectively. The slight shifts are negligible, confirming that Rh stays in its metallic form throughout HER testing. Meanwhile, in the Rh1c/Co_3_O_4_-@GP composite Figure 6B), the Rh 3d spectrum shows clear peaks at ∼307.3 eV and ∼312.0 eV, corresponding to Rh 3d_5/2_ and 3d_3/2_, respectively. These match well with those of metallic Rh^0^. There are no noticeable changes seen after voltammetric cycling, indicating Rh remains in its zerovalent state and is chemically stable under HER conditions. The Co 2p spectra were also evaluated in Figure 6C and show typical features of mixed-valence cobalt oxides, with Co 2p_3/2_ and 2p_1/2_ peaks and their satellite signals. Deconvolution reveals both Co^2+^ and Co^3+^ species.^57^ The Co^3+^ 2p_3/2_ peaks appear around 779.3 eV (before the HER stability experiment) and 779.2 eV (after the HER stability experiment), while Co^2+^ 2p_3/2_ appears near 780.9 eV. The corresponding 2p_1/2_ satellite peaks follow a similar trend. The Co^2+^:Co^3+^ ratio is close to 1:2 in both cases, matching the characteristics of the Co_3_O_4_ phase. These results ensure that Co_3_O_4_ is the main cobalt oxide present and that its oxidation state remains unchanged, even after HER operation. Overall, the XPS results demonstrate the structural and chemical stability of both Co_3_O_4_ and metallic Rh during electrocatalysis, supporting their role in maintaining a high HER performance.

**Figure 6:**
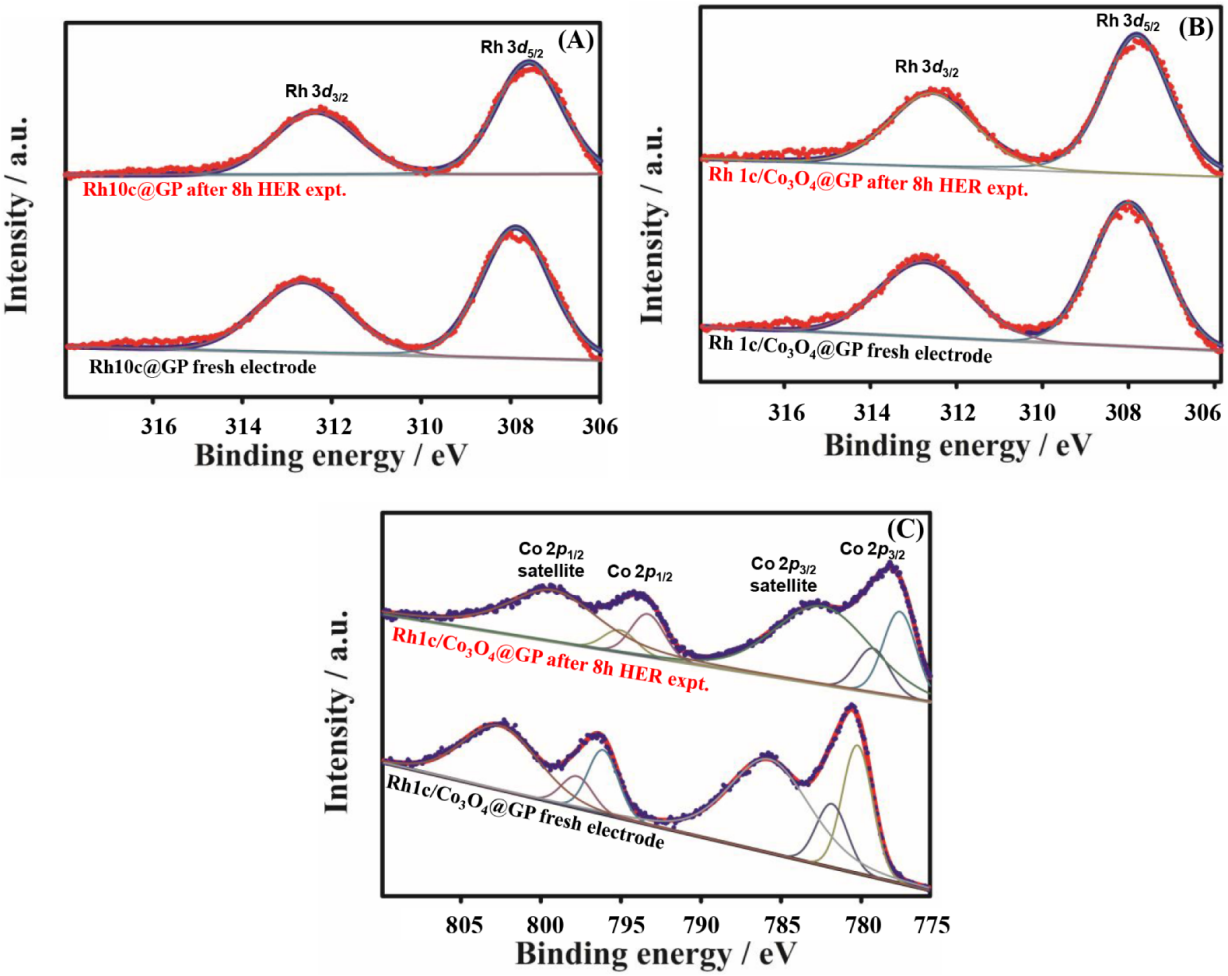
High-resolution XPS spectra after deconvolution of Rh of (A) the Rh10c-@GP electrode and (B) the Rh1c/Co_3_O_4_@GP electrode in the Rh *3d* regions. (C) Rh1c/Co_3_O_4_@GP electrode in the Co 2p regions.

### 3.7 Relative HER Performance

Cobalt-based materials were used as promising electrocatalysts for HER due to their higher percentage of natural abundance, significant catalytic properties, stability, and relatively low cost. According to recent reviews, cobalt oxides and their derivatives typically exhibit HER overpotentials from 80 to 200 mV at a current density of 10 mA/cm^2^, with corresponding Tafel slopes between 50 and 120 mV/dec, particularly when supported on materials with high conductivity, such as reduced graphene oxide (rGo) or carbon nanotubes (CNTs).^57,61,62^ Electrodeposited cobalt-oxide thin films, while effective, often show overpotentials in the 100–150 mV range and moderate long-term stability. Some improvements have been achieved by incorporating nanostructured supports or heterostructures, but performance limitations remain.^61^

In comparison, the electrode developed in our study, comprising cobalt oxides and a trace amount of Rh deposited onto a GP surface, exhibits a significantly enhanced HER activity. It delivers a low overpotential of just 44 mV at 10 mA/cm^2^, a Tafel slope of 39 mV/dec, and maintains stable performance over an 8-h testing period. These results surpass many reported Co-based systems, including Ni–Co/graphite electrodes with overpotentials 255 mV and Co-rGo hybrids with tafel Slope ∼75 mV/dec.^62,63^ The superior catalytic behavior is likely because of the synergistic interplay between Co and Rh, which improves electron-transfer efficiency and lowers the energy barrier for hydrogen evolution. Additionally, the use of pencil graphite offers a simple, low-cost, and conductive platform that supports uniform catalyst deposition. Together, these attributes make the proposed electrode a compelling alternative to conventional non-noble systems and a cost-effective competitor to noble-metal-based HER catalysts. Several data points pertinent to HER activity with various electrodes are provided in Table 2.

**Table 2:** Comparative Electrochemical Performance of the Rh1c/Co_3_O_4_-@GP Catalyst with Reported Cobalt-Based HER Systems

Reference	Catalyst type	Substrate support	Overpotential (η_10_)@10 mA/cm^2^	Tafel slope	Stability
This work	Co oxides + trace Rh	GP	44 mV	39 mV/dec	8 h
^59^	Co oxides, sulfides	Mixed	100–150 mV	60–120 mV/dec	Moderate
^60^	Co–N_*x*_/C, Co-rGO	rGO, CNT	80–150 mV	50–100 mV/dec	Long-term
^61^	Co hydroxides, oxides	Glassy carbon	∼150 mV	70–100 mV/dec	Moderate
^57^	Mo, RuCo, PdAu	Graphene, foams	80–150 mV	30–80 mV/dec	Excellent
^62^	Ni:Co alloy	Graphite rod	255 mV	–	20 h
^63^	Co + Co_3_O_4_ on rGO	GCE	∼100 mV	75 mV/dec	Long-term

## 4 Conclusions

In our pursuit of developing cost-effective and high-performance electrocatalysts for the HER in acidic environments, we have successfully fabricated a graphite electrode modified with a thin film of Co_3_O_4_ supported by trace amounts of Rh. This Rh1c/Co_3_O_4_-@GP electrode, prepared using an electrodeposition method, exhibited outstanding catalytic performance for HER with long-term stability. Compared to both bare graphite and a graphite electrode modified with a higher Rh content (10 cycles), our new electrode system significantly reduced the overpotential and showed a lower Tafel slope, indicating enhanced catalytic activity. The electrode demonstrated excellent long-term stability in acidic conditions. The hydrogen produced through this method can serve as a clean fuel, offering a sustainable solution that avoids carbon dioxide emissions and supports a greener energy future. Finally, the fabricated electrode may be an alternative of Pt/C electrode which is widely used as the reference HER catalyst.

## Supplementary Material



## Notes

Linear polarization curves for bare GP, Co_3_O_4_@GP, Rh1c@GP and Rh1c/Co_3_O_4_@GP electrodes; Nyquist plots of different electrodes; table showing HER kinetic performance of the Rh/Co_3_O_4_@GP electrode at varying Rh loading; cyclic voltammograms for bare GP, Co_3_O_4_@GP, Rh10c@GP, and Rh1c/Co_3_O_4_@GP electrodes at different scan rates; schematic electrochemical setup used for electrodeposition (PDF)
