# Supplementary material for: Superb Enhancement of Hydrogen Evolution in an Acidic Medium over a Cobalt Oxide Surface with Trace Incorporation of Rhodium Particles
Source: ACS Omega. 2025 Dec 18;11(1):1461–71. doi: 10.1021/acsomega.5c08740 (PMC12809353; doi:10.1021/acsomega.5c08740)
Supplement: Supplementary file 1 [file ao5c08740_si_001.pdf]

Supporting Information

Superb enhancement of hydrogen evolution in an acidic medium over cobalt oxide surface with a trace incorporation of rhodium particles

Kazi Hamidur Rashid [a], Mohammad Imran Hossain [a], Md Abdul Malek [b], Mohammad Afsar Uddin [f,*], Kentaro Aoki [c], Yuki Nagao [c], Nayan Ranjan Singha [d], Mostafizur Rahaman [e], Merajuddin Khan [e], Mohammad A. Hasnat [a,*]

[a] *Electrochemistry & Catalysis Research Laboratory (ECRL), Department of Chemistry, School of Physical Sciences, Shahjalal University of Science and Technology, Sylhet-3114, Bangladesh*

[b] *The University of Alabama, Tuscaloosa, Alabama-35401, United States of America*

[c] *School of Materials Science, Japan Advanced Institute of Science and Technology, Nomi, Ishikawa 923-1292, Japan*

[d] *Advanced Polymer Laboratory, Department of Polymer Science and Technology, Government College of Engineering and Leather Technology (Post-Graduate), Kolkata-700106, West Bengal, India*

[e] *Department of Chemistry, College of Science, King Saud University, P.O. Box 2455, Riyadh 11451, Saudi Arabia*

[f] *Instituto de Ciencia de Materiales de Madrid (ICMM), CSIC, C / Sor Juana Ines de la Cruz 3, Madrid 28049, Spain.*

Corresponding author: Mohammad A. Hasnat; Mohammad Afsar Uddin

E-mail address: mah-che@sust.edu; m.auddin@csic.es

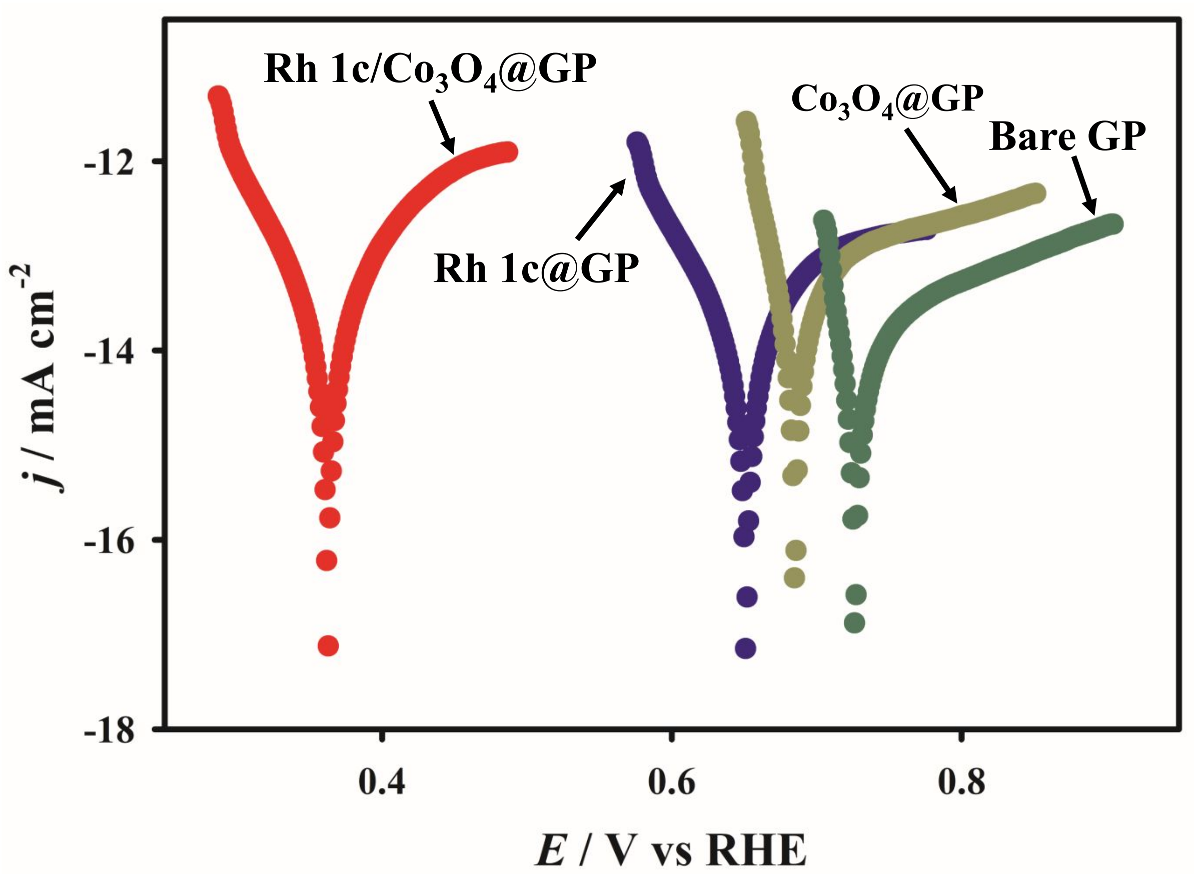

Figure S1. Linear polarization curves recorded in 0.5 M H_2SO_4 solution using bare GP, Co_3O_4@GP, Rh 1c@GP and Rh1c/Co_3O_4@GP electrodes at a scan rate of 0.1 Vs^{-1}.

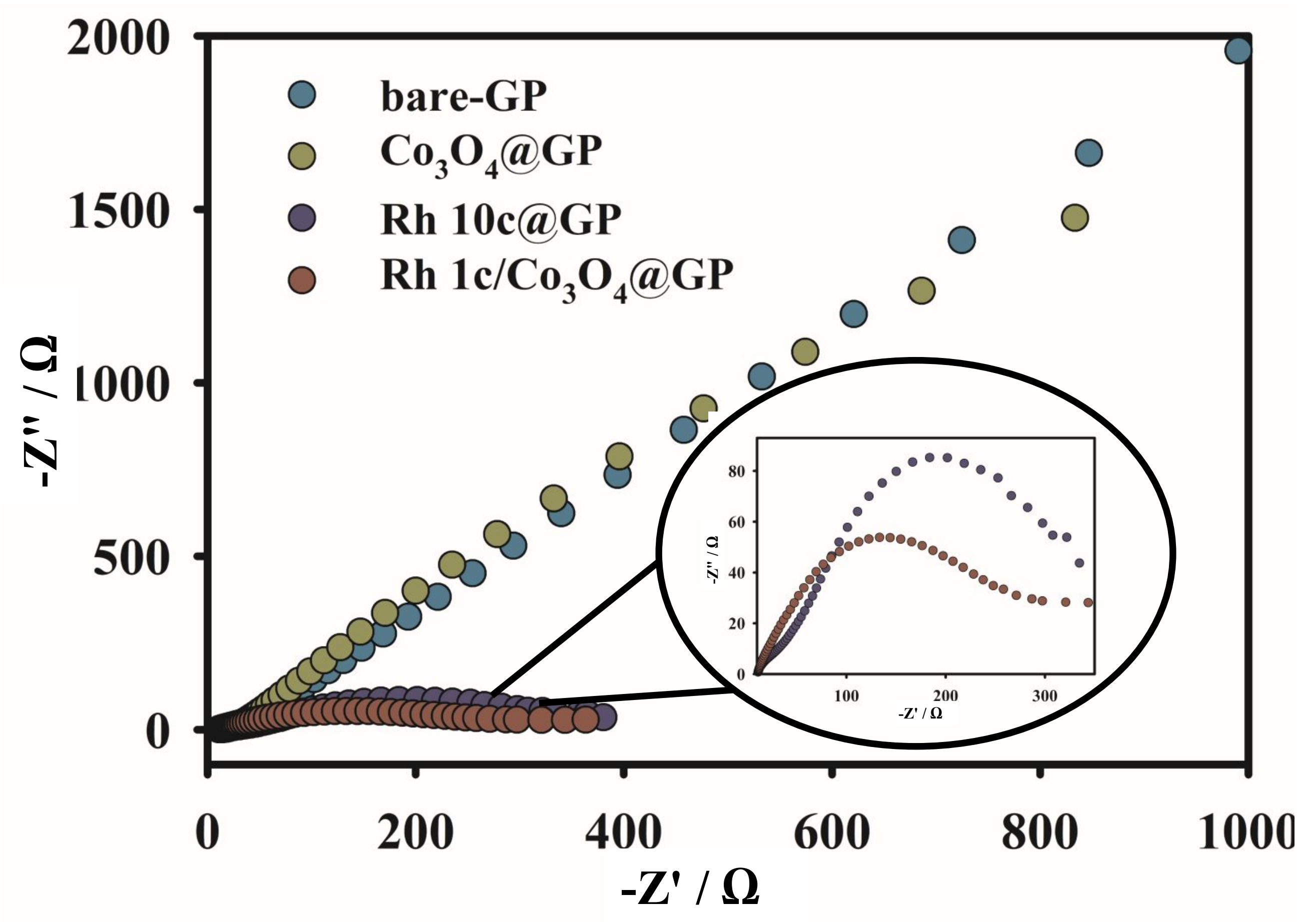

Figure S2. Nyquist plots of different electrodes for HER performance in 0.5 M H_2SO_4.

Table S1. Summary of the HER kinetic performance of the Rh/Co_3O_4@GP electrode at varying Rh loading.

Electrode	Amount of deposition /μg cm^{-2}	b_T / mV dec^{-1}	η_{10} / mV	E_{onset} / mV
**Rh 1c / Co_3O_4@GP**	2.792	39	43	10.7
**Rh 2c / Co_3O_4@GP**	5.328	118	49.7	11.3
**Rh 3c / Co_3O_4@GP**	7.536	120	52.2	11.3
**Rh 4c / Co_3O_4@GP**	10.168	123	56.9	16.3
**Rh 5c / Co_3O_4@GP**	12.550	56	152.5	55.2
**Rh10c / Co_3O_4@GP**	24.592	93	255	241.5

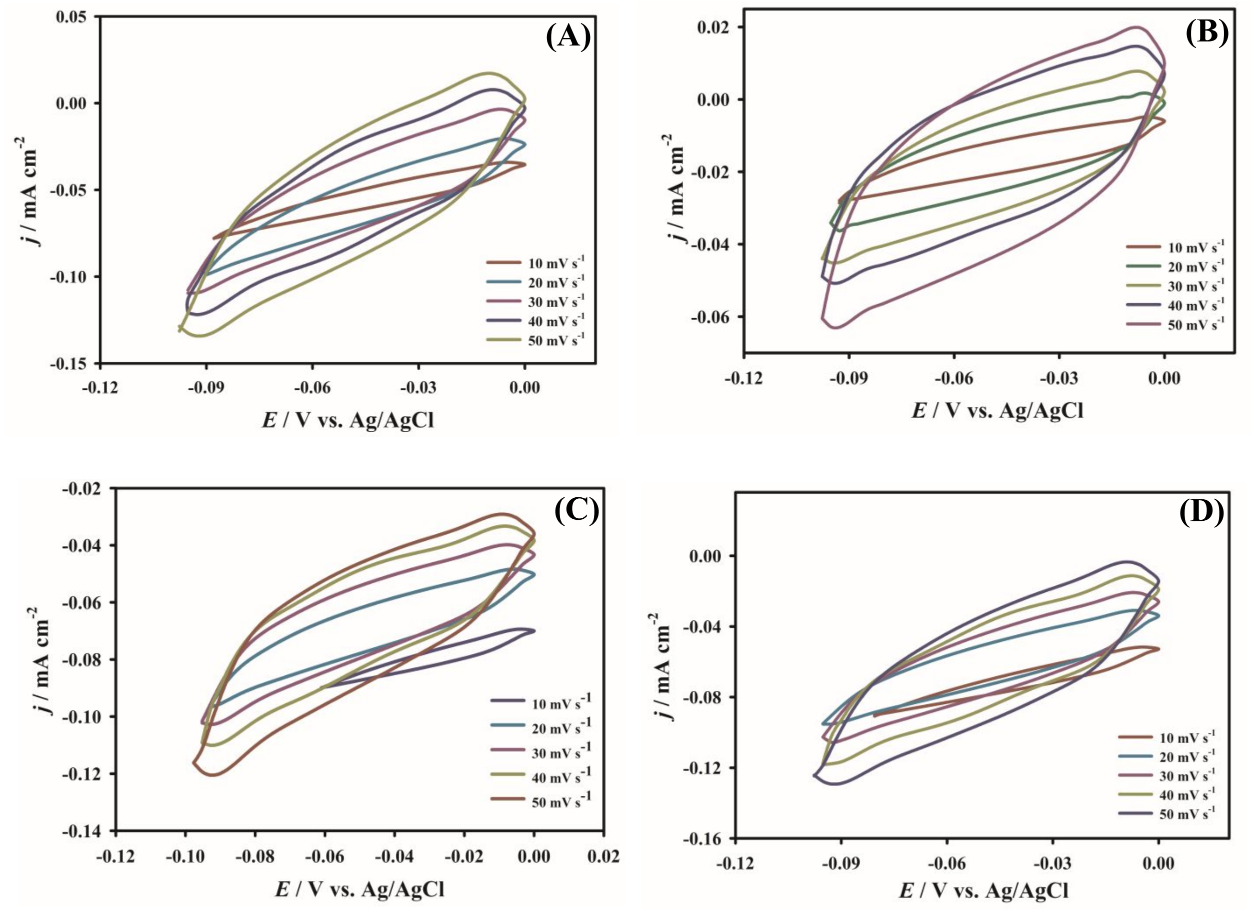

Figure S3. Cyclic voltammograms at different scan rates for (A) bare-GP, (B) Co_3O_4@GP, (C) Rh 10c@GP, and (D) Rh 1c/Co_3O_4@GP electrodes.

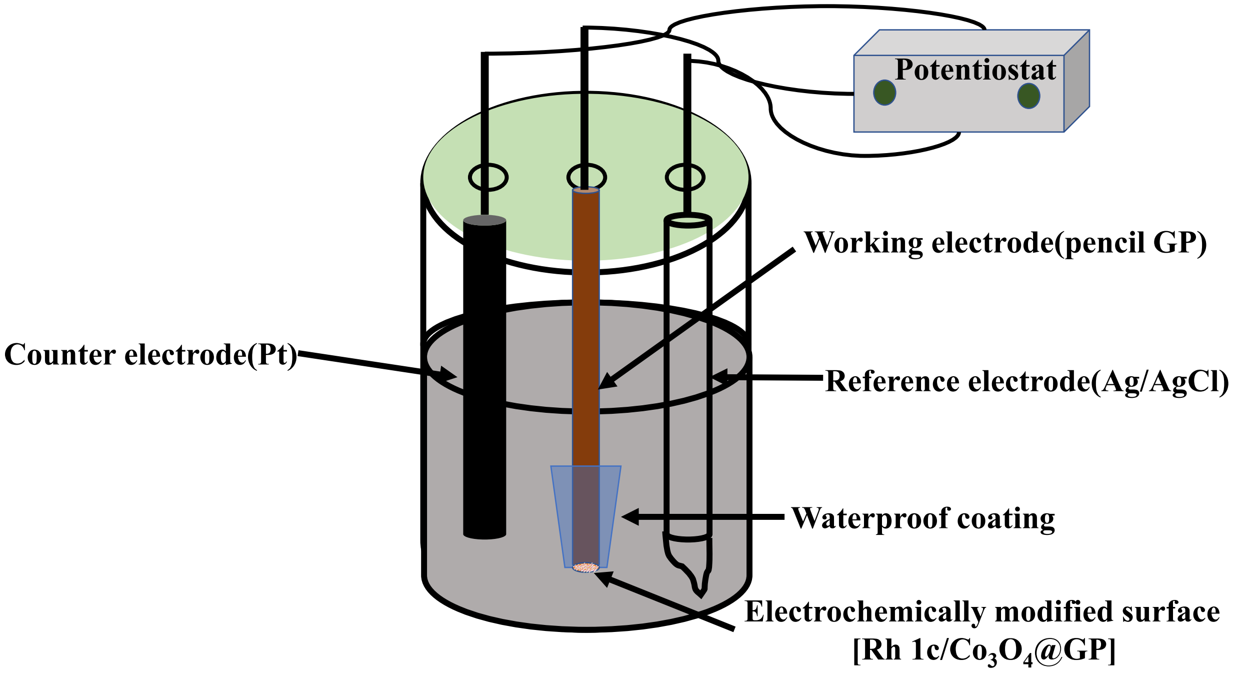

Figure S4. Schematic electrochemical setup used for electrodeposition on modified electrode.
